# Cardiogenic Shock Secondary to Dynamic Left Ventricular Outflow Tract Obstruction and Apical Ballooning after Nonmitral Cardiovascular Surgery

**DOI:** 10.1155/2020/8826187

**Published:** 2020-11-24

**Authors:** Hoang Bac Nguyen, Hoang Dinh Nguyen, Thi Thanh Thuy Tran, Minh Khoi Le

**Affiliations:** ^1^Department of General Surgery, University Medical Center, University of Medicine and Pharmacy at Ho Chi Minh City, 215 Hong Bang Str., Ward 11, District 5, Ho Chi Minh City, Vietnam; ^2^Cardiovascular Center, University Medical Center, University of Medicine and Pharmacy at Ho Chi Minh City, 215 Hong Bang Str., Ward 11, District 5, Ho Chi Minh City, Vietnam

## Abstract

**Background:**

The dynamic obstruction of the left ventricular outflow tract (LVOT) is a well-known complication in mitral annuloplasty but rarely seen in nonmitral cardiovascular surgery. The dynamic LVOT obstruction can lead to hemodynamic instability, even shock and the treatment is significantly different from the standard approach. *Case Presentation*. We reported a case of low cardiac output syndrome (LCOS) with severe mitral regurgitation (MR), dramatically reduced left ventricular ejection fraction (LVEF) after coronary artery bypass grafting in a 72-year-old female requiring an escalation of inotropic support, volume restriction, and mechanical support. The detailed echocardiography combined with lung ultrasound revealed a dynamic systolic anterior movement of the anterior mitral leaflet (SAM), apical ballooning, and no significant lung congestion. Intravenous fluids were given, diuretics withdrawn, inotrope discontinued, and vasopressors uptitrated. The dynamic SAM was rapidly relieved, the hemodynamics was stabilized, and the LVEF was improving. The patient was discharged in good condition without residual LVOT obstruction and trace MR.

**Conclusion:**

We strongly suggest that a detailed echocardiography should be performed in any patient who presents in shock to rule out a dynamic LVOT obstruction. Lung ultrasound should be a routine examination in addition to echocardiography. Once SAM is detected, treatment should be based on volume expansion, inotrope discontinuation, and a careful afterload increasing.

## 1. Background

The incidence of dynamic systolic anterior movement of the anterior mitral leaflet (SAM) leading to left ventricular outflow tract (LVOT) obstruction after mitral annuloplasty was reported to range between 1 and 16% [1]. In contrast, SAM in major nonmitral cardiovascular surgery is a rare condition [2, 3] but could theoretically worsen an unstable hemodynamics or even trigger a refractory cardiogenic shock. Prompt recognition of this rare presentation is of clinical importance because vasodilators and inotropic agents used in cardiogenic shock can worsen hemodynamics in such patients [4]. In a large series of SAM after mitral valve repair, Brown and coworkers suggested that most cases of SAM resolved with conservative measures including beta-blockade, vasoconstriction, and fluid administration. The intravenous *β* blocker, aggressive volume expansion was used with high success in 3 patients who developed an “isolated” LVOT obstruction [5]. The decision to withhold inotropic support, administer fluid, and add beta-blockade might be hard to make, especially in the context of poor ventricular contraction and refractory hypotension. A combination of echocardiography and lung ultrasound to correctly point out the primary etiology of hemodynamic instability and investigate the backward consequences on pulmonary circulation might be a reasonable clinical approach. In this paper, we reported a case of severe SAM after coronary artery bypass grafting (CABG) with low left ventricular ejection fraction (LVEF), which was promptly diagnosed and successfully treated based on echocardiography and lung ultrasound.

## 2. Case Presentation

**Figure 1:**
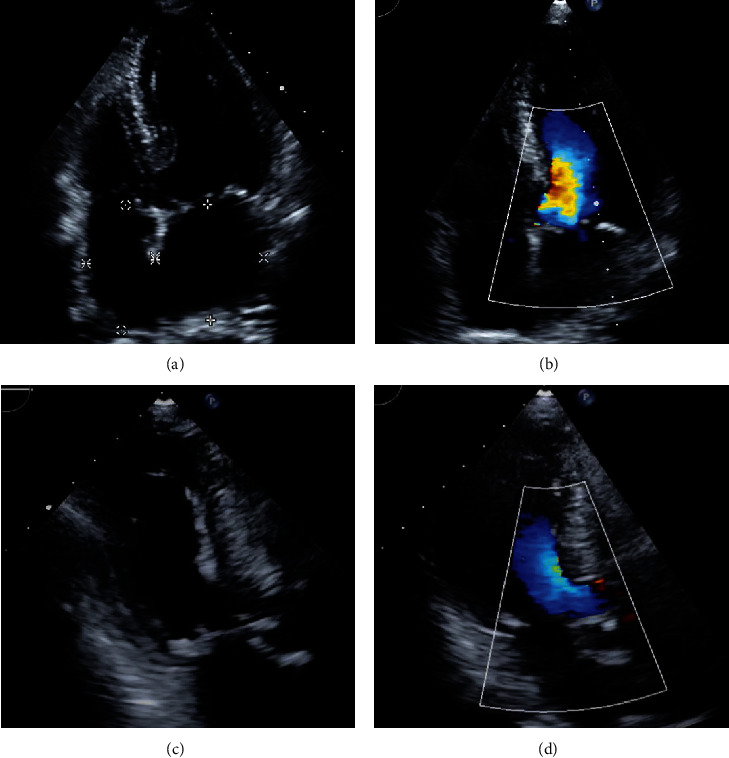
The preoperative echocardiogram was showing no obstruction of the left ventricular outflow tract. (a) The apical four-chamber view. (b) The apical four-chamber view with flow mapping. (c) The apical three-chamber view. (d) The apical three-chamber view with flow mapping. A normal left ventricular contraction and mild aortic regurgitation was noted on the apical three-chamber view (Supplement video 1).

A 72-year-old Vietnamese woman presented to a community hospital due to chest pain for the previous three days. The medical history was remarkable, with hypertension and dyslipidemia lasting for seven years. ECG confirmed a non-ST-segment elevation myocardial infarction. Coronary angiography showed a left main coronary artery disease and two-vessel disease. The patient was transferred to our center for an elective on-pump CABG surgery. The preoperative echocardiography showed good biventricular contraction with neither regional wall motion abnormality nor LVOT obstruction (Figure 1 and Supplement video 1). Cavoatrial (two-stage) cannulation and antegrade cardioplegia with Custodiol solution were performed according to our institutional protocol. The surgery progressed uneventfully, and the patient was hemodynamically stable during the procedure. On admission to the cardiac intensive care unit, the heart rate increased to 135 bpm, blood pressure (BP) varied in a wide range with systolic BP from 90 to 125 mmHg, and serum lactate was 2.8 mmol/L. A quick cardiac ultrasound scan showed a severely reduced LVEF of around 30% and a significant mitral regurgitation, which had been not noticed on preoperative echocardiography.

**Figure 2:**
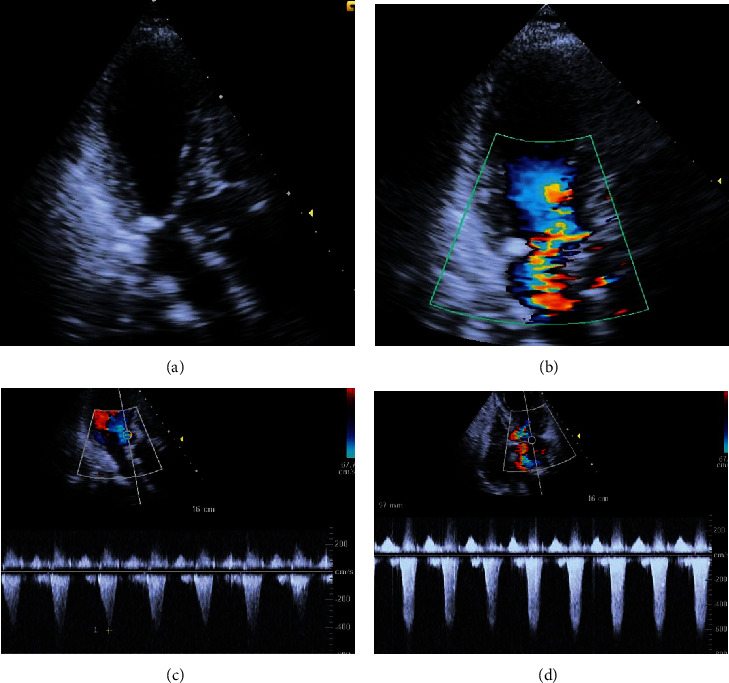
The postoperative echocardiogram performed during hemodynamic instability revealed a left ventricular outflow tract (LVOT) obstruction caused by systolic anterior movement of the mitral leaflet (SAM). (a) SAM leading to dynamic LVOT obstruction and the apical ballooning of the left ventricle. (b) A severe mitral regurgitation secondary to SAM. (c) Elevated pressure gradient across LVOT of 73 mmHg with typical dagger-shaped spectral Doppler. (d) High pressure gradient across the mitral valve in systole. The SAM and ballooning of the LV apex was seen on the apical three-chamber view (Supplement video 2).

**Figure 3:**
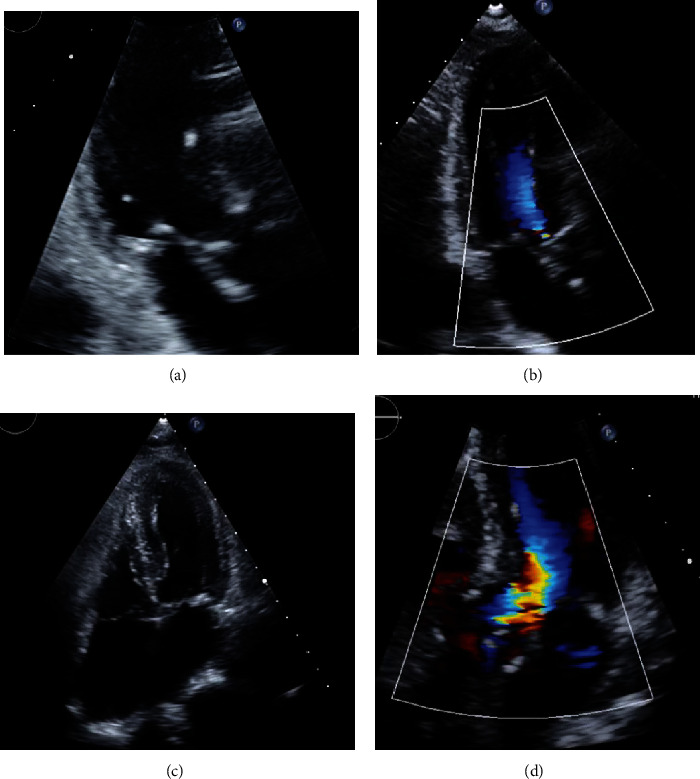
The echocardiogram performed on 4^th^ day postoperatively showed complete relief of left ventricular outflow tract (LVOT) obstruction. (a) No SAM was seen on the three-chamber view. (b) No flow acceleration nor mitral regurgitation on the three-chamber view with color mapping. (c) Patent LVOT and regular LV shape. (d) No flow acceleration nor mitral regurgitation on the five-chamber view with color mapping. The Supplement video 3 and Supplement video 4 provide a better appreciation of normal LV shape, good LV contraction, and patent LVOT.

The intra-aortic balloon pump (IABP) was inserted, dobutamine was increased from 5 to 10 mcg/kg/min, norepinephrine was added at a dose of 0.025 mcg/kg/min, and repeated doses of intravenous furosemide were administered. The clinical and hemodynamic picture continued to deteriorate. A senior cardiologist was called upon to perform a comprehensive echocardiography, which revealed a poor left ventricular contraction with LVEF of 28% (biplane Simpson method), a severe LVOT obstruction with a pressure gradient of 73 mmHg due to an obvious SAM, and a significant mitral regurgitation secondary to SAM (Figure 2 and Supplement video 2). Of note, significant mid and apical hypokinesia and nearly normal basal contraction were observed. The management plan would be fluid expansion, inotrope reduction or discontinuation, and cautious vasopressor uptitration, among other adjustments. Since the left ventricular systolic function was poor, and the mitral regurgitation was severe, pulmonary edema might be engendered, which contradicted the abovementioned approach. Lung ultrasound was performed, showing an A-profile ruling out significant pulmonary congestion. Subsequently, 500 ml normal saline was given over 45 minutes, dobutamine was discontinued, and norepinephrine was increased to 0.1 mcg/kg/min. All these interventions were carried out under close hemodynamic monitoring, echocardiography, and lung ultrasound. The LVOT obstruction was rapidly relieved, mitral regurgitation became obviously less pronounced, and the patient's hemodynamics gradually but steadily improved overnight. The IABP was withdrawn two days after insertion. The patient was extubated one day afterward. During these days, LVOT obstruction was no longer noticed on echocardiography. Parallel, the MR reversed from severe to mild degree as being observed preoperatively, and the LVEF gradually returned to normal (Figure 3). The patient was discharged with an excellent functional status, LVEF of 51%, no apical ballooning of the left ventricle, trace MR, and no LVOT obstruction (Supplement video 3 and Supplement video 4).

## 3. Discussion

In contrast to the SAM after mitral valve repair, which is a relatively common postoperative manifestation [1, 6], SAM that occurs after nonmitral cardiovascular surgery is still an exceptional entity [2, 3] and more likely to be missed or not quickly detected as the primary cause of hemodynamic instability. In a patient who underwent a CABG surgery, the principal causes of hypotension would be poor contractility due to cardiac stunning or graft flow abnormality. The first cardiac scan did not reveal a SAM even though poor LV contraction and severe mitral regurgitation was correctly noted. The regrettable misdiagnosis underlines the importance of an exhaustive echocardiography in a patient with refractory hypotension, and no shortcut should be tolerated.

It has been sporadically reported that dynamic SAM occurred in patients with acute coronary syndrome [7], sepsis [8], and severe anemia [3]. Said and coworkers suggested that the thickening of the interventricular septum (bulging subaortic septum) was one of the most important factors of dynamic LVOT obstruction after mitral valve repair [9]. Our patient did show some degree of bulging subaortic septum with minimal velocity acceleration of blood flow in LVOT on preoperative echocardiography. This anatomic feature might predispose the patient to the development of SAM in addition to other factors. To and coworkers suggested that the use of inotropes in this situation would be likely to worsen the LVOT obstruction and MR [10]. Our initial management with increased dobutamine dose repeated intravenous boluses of furosemide would worsen the patient's hemodynamics. Interestingly, in our patient, echocardiography clearly detected an apical hypokinesia with nearly normal basal contraction. It was reported that SAM has been observed in Takotsubo cardiomyopathy [10, 11]. El Mahmoud and coworkers [12] found that the prevalence of septal bulge was 100% in patients with Takotsubo cardiomyopathy and LVOT obstruction versus 29% in patients without LVOT obstruction (*P* = 0.002). Coronary microcirculation impairment is another predisposing factor for apical ballooning [13]. In our patient, who had suffered a chronic coronary disease, coronary microcirculation was undoubtedly impaired. The antegrade cardioplegia might not protect the myocardium optimally, leading to acute myocardial stunning. In brief, the causes of apical ballooning, LVOT obstruction, and severe MR in our patient were multifactorial. If this dynamic SAM had been not detected early, the clinical deterioration would have accentuated due to improper management.

Landoni and coworkers recommended the stepwise management for SAM after mitral valve repair: expanding intravascular volume, discontinuing all inotropic drugs, and increasing the afterload [14]. The therapeutic approach in patients with SAM not associated with mitral valve repair appears similar [1]. The management of patients with Takotsubo cardiomyopathy with acute hemodynamic instability does not significantly differ from that in patients with dynamic LVOT obstruction. However, there remain controversies over the use of cardiac stimulants and diuretics in patients with Takotsubo cardiomyopathy [15], whereas these agents are contraindicated in patients with dynamic LVOT obstruction. In our patient, the volume expansion, withdrawal of inotropic support, and increase of vasopressor were planned. However, the poor LV contraction, which was a rare presentation in SAM, would hinder this approach due to the concern of lung congestion or even an acute pulmonary edema. We additionally performed a lung ultrasound, which showed no predominant B-lines, ruling out a significant lung congestion. With the information from both echocardiography and lung ultrasound, we decided to expand intravascular volume cautiously, discontinue dobutamine and furosemide, and increase intravenous norepinephrine dosage. This management led to rapid and complete relief of LVOT obstruction, reversal of severe mitral regurgitation, and stabilization of hemodynamics.

Our case report represents a rare cause of hemodynamic instability in a patient undergoing CABG with a particular manifestation of LVOT obstruction, including dramatic LVEF reduction in association with an apical ballooning, severe MR. Echocardiography and lung ultrasound played crucial in detecting SAM as the principal etiology of shock. This finding totally changed the therapeutic approach and led to the successful management.

## 4. Conclusion

We reported a case of systolic anterior movement of the anterior mitral leaflet with unusual clinical manifestation. This case highlighted the critical role of combining echocardiography and lung ultrasound in diagnosis and treatment. The SAM after nonmitral cardiovascular surgery is a rare but real pathological entity and may be triggered by multiple factors. The management of dynamic LVOT obstruction and MR caused by SAM is quite different from and somehow opposite to the therapeutic approach to common causes of postsurgical LCOS such as myocardial stunning, other complications of inappropriate myocardial protection, or problems of coronary artery flow. A prompt and correct diagnosis of SAM plays a pivotal role in establishing a proper treatment. A comprehensive echocardiography is required in patients with postsurgical hemodynamic instability and should not focus merely on ventricular contraction. Lung ultrasound should be performed to complete the patient's hemodynamic picture and help to titrate carefully the treatment, especially the decision of volume expansion. Treatment of LVOT obstruction caused by SAM is based on intravascular volume expansion, discontinuation of inotropic support, and increase of systemic vascular resistance. In the majority of cases, SAM is reversed, LVOT obstruction is relieved, MR is significantly reduced, and hemodynamics rapidly improves.

## Data Availability

The essential data are provided in the manuscript and Supplement materials. There is no additional data or material.

## Abbreviations

CABG: Coronary artery bypass grafting
IABP: Intra-aortic balloon pump
LCOS: Low cardiac output syndrome
LVEF: Left ventricular ejection fraction
LVOT: Left ventricular outflow tract
MR: Mitral regurgitation
SAM: Systolic anterior movement of the anterior mitral leaflet.

## Supplementary Materials

Supplementary 1 Supplement video 1: the preoperative three-chamber view with color Doppler showed an excellent left ventricular contraction, no left ventricular outflow tract obstruction, no significant mitral regurgitation, and a mild aortic regurgitation.

Supplementary 2 Supplement video 2: the postoperative echocardiogram performed during hemodynamic instability revealed a dynamic left ventricular outflow tract obstruction caused by systolic anterior movement of the mitral leaflet. Of note, the LV systolic contraction was severely reduced in apical and mid segments, while the basal parts were hyperdynamic (apical ballooning).

Supplementary 3 Supplement video 3: the echocardiogram performed on 4^th^ day postoperatively showed a complete recovery with a regular left ventricular shape, good contraction, no left ventricular outflow tract obstruction, and no mitral regurgitation.

Supplementary 4 Supplement video 4: the echocardiogram performed on 4th day postoperatively showed an excellent left ventricular systolic function, a regular movement of the mitral leaflet as well as a patent left ventricular outflow tract.
